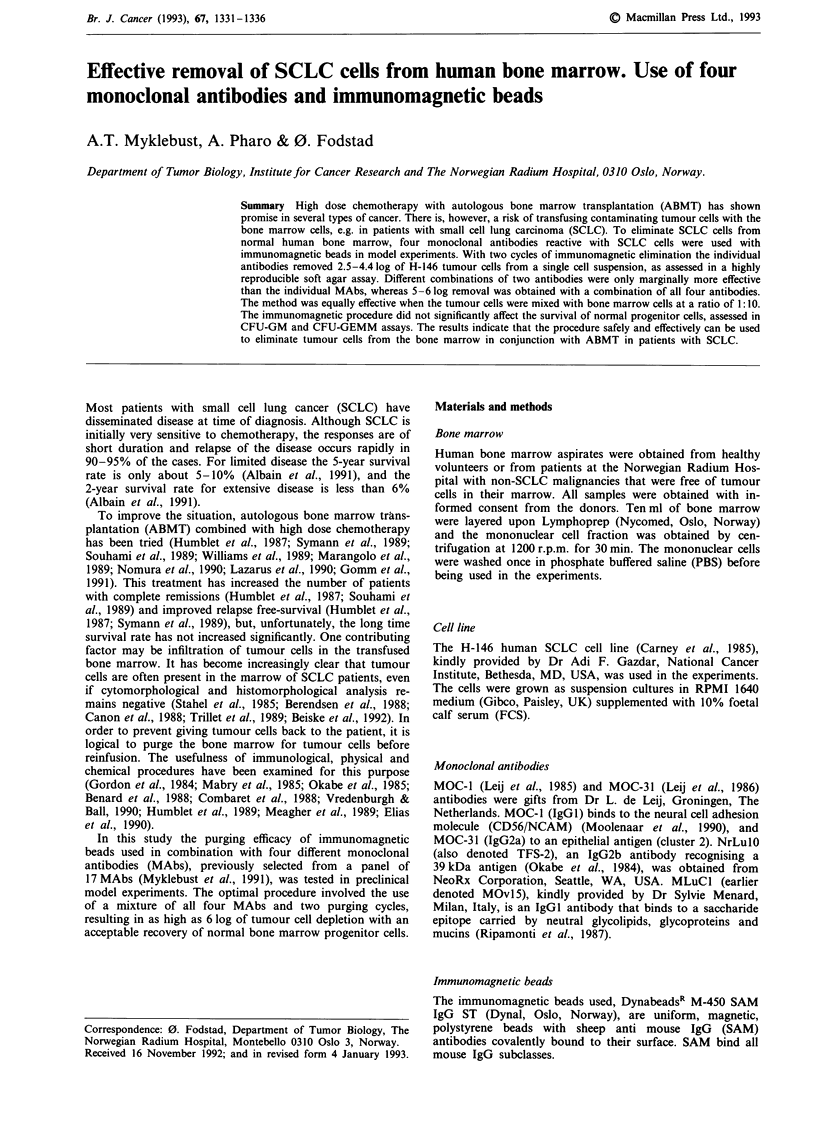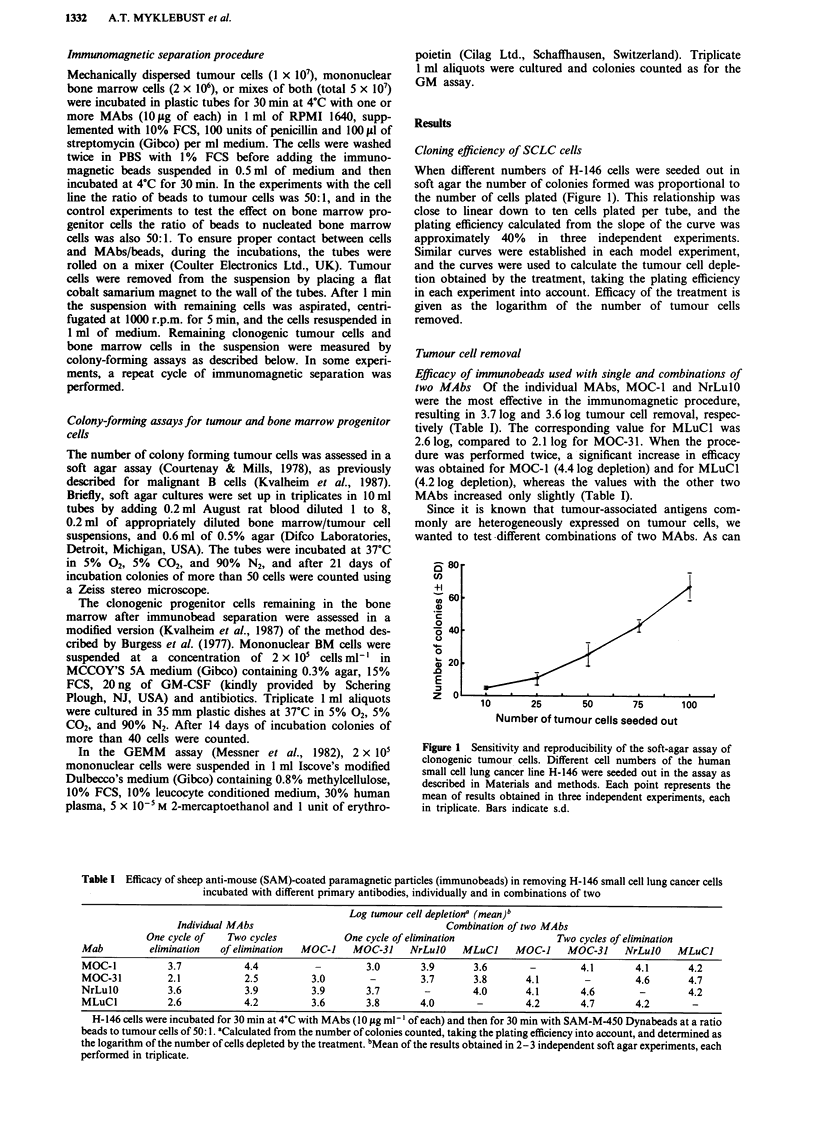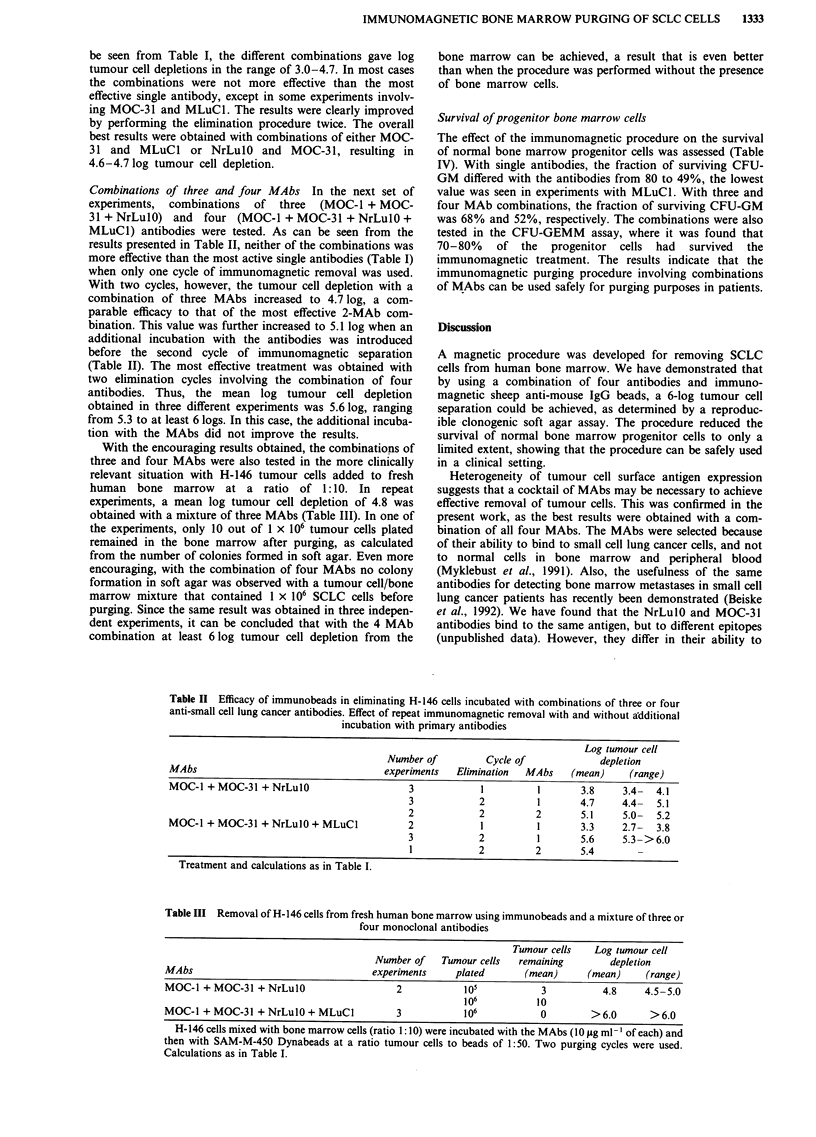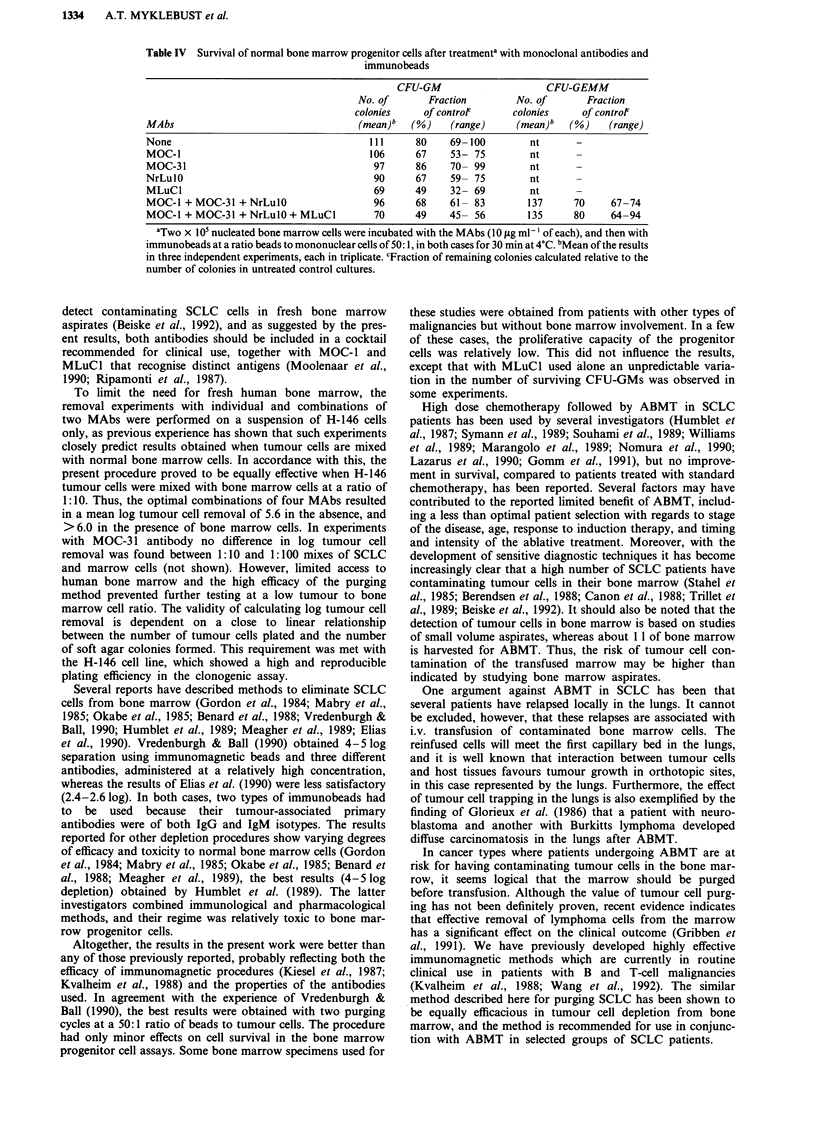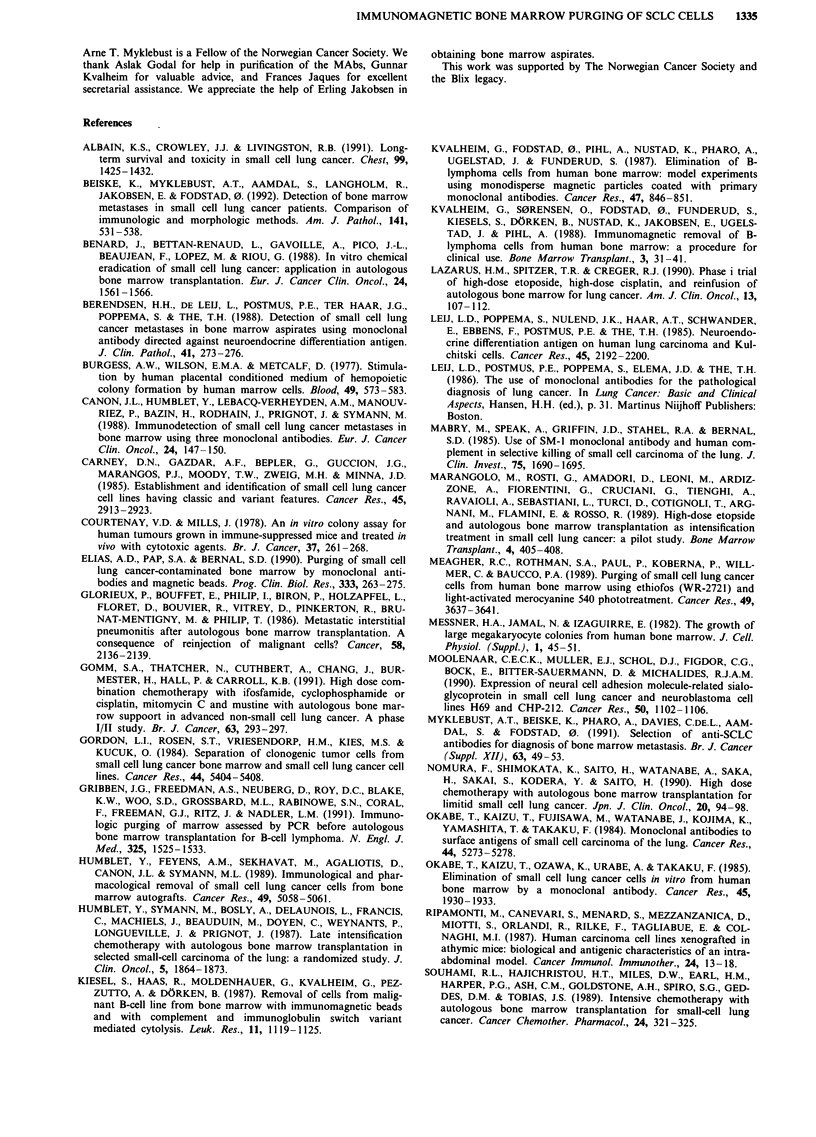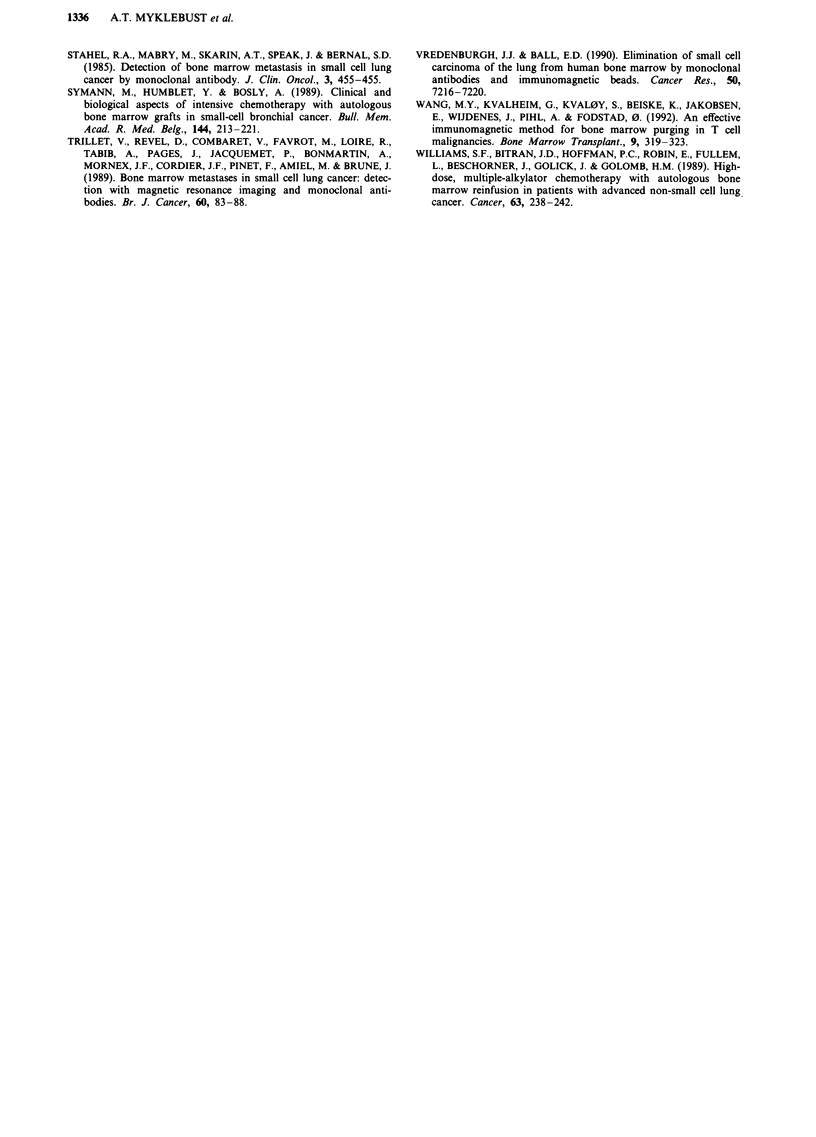# Effective removal of SCLC cells from human bone marrow. Use of four monoclonal antibodies and immunomagnetic beads.

**DOI:** 10.1038/bjc.1993.246

**Published:** 1993-06

**Authors:** A. T. Myklebust, A. Pharo, O. Fodstad

**Affiliations:** Department of Tumor Biology, Institute for Cancer Research, Oslo, Norway.

## Abstract

High dose chemotherapy with autologous bone marrow transplantation (ABMT) has shown promise in several types of cancer. There is, however, a risk of transfusing contaminating tumour cells with the bone marrow cells, e.g. in patients with small cell lung carcinoma (SCLC). To eliminate SCLC cells from normal human bone marrow, four monoclonal antibodies reactive with SCLC cells were used with immunomagnetic beads in model experiments. With two cycles of immunomagnetic elimination the individual antibodies removed 2.5-4.4 log of H-146 tumour cells from a single cell suspension, as assessed in a highly reproducible soft agar assay. Different combinations of two antibodies were only marginally more effective than the individual MAbs, whereas 5-6 log removal was obtained with a combination of all four antibodies. The method was equally effective when the tumour cells were mixed with bone marrow cells at a ratio of 1:10. The immunomagnetic procedure did not significantly affect the survival of normal progenitor cells, assessed in CFU-GM and CFU-GEMM assays. The results indicate that the procedure safely and effectively can be used to eliminate tumour cells from the bone marrow in conjunction with ABMT in patients with SCLC.


Br. J. Cancer (1993), 67, 1331–1336 © Macmillan Press Ltd., 1993

### Effective removal of SCLC cells from human bone marrow. Use of four monoclonal antibodies and immunomagnetic beads

A.T. Myklebust, A. Pharo & Ø. Fodstad

*Department of Tumor Biology, Institute for Cancer Research and The Norwegian Radium Hospital, 0310 Oslo, Norway.*

**Summary** High dose chemotherapy with autologous bone marrow transplantation (ABMT) has shown promise in several types of cancer. There is, however, a risk of transfusing contaminating tumour cells with the bone marrow cells, e.g. in patients with small cell lung carcinoma (SCLC). To eliminate SCLC cells from normal human bone marrow, four monoclonal antibodies reactive with SCLC cells were used with immunomagnetic beads in model experiments. With two cycles of immunomagnetic elimination the individual antibodies removed 2.5–4.4 log of H-146 tumour cells from a single cell suspension, as assessed in a highly reproducible soft agar assay. Different combinations of two antibodies were only marginally more effective than the individual MAbs, whereas 5–6 log removal was obtained with a combination of all four antibodies. The method was equally effective when the tumour cells were mixed with bone marrow cells at a ratio of 1:10. The immunomagnetic procedure did not significantly affect the survival of normal progenitor cells, assessed in CFU-GM and CFU-GEMM assays. The results indicate that the procedure safely and effectively can be used to eliminate tumour cells from the bone marrow in conjunction with ABMT in patients with SCLC.

Most patients with small cell lung cancer (SCLC) have disseminated disease at time of diagnosis. Although SCLC is initially very sensitive to chemotherapy, the responses are of short duration and relapse of the disease occurs rapidly in 90–95% of the cases. For limited disease the 5-year survival rate is only about 5–10% (Albain *et al.*, 1991), and the 2-year survival rate for extensive disease is less than 6% (Albain *et al.*, 1991).

To improve the situation, autologous bone marrow transplantation (ABMT) combined with high dose chemotherapy has been tried (Humblet *et al.*, 1987; Symann *et al.*, 1989; Souhami *et al.*, 1989; Williams *et al.*, 1989; Marangolo *et al.*, 1989; Nomura *et al.*, 1990; Lazarus *et al.*, 1990; Gomm *et al.*, 1991). This treatment has increased the number of patients with complete remissions (Humblet *et al.*, 1987; Souhami *et al.*, 1989) and improved relapse free-survival (Humblet *et al.*, 1987; Symann *et al.*, 1989), but, unfortunately, the long time survival rate has not increased significantly. One contributing factor may be infiltration of tumour cells in the transfused bone marrow. It has become increasingly clear that tumour cells are often present in the marrow of SCLC patients, even if cytomorphological and histomorphological analysis remains negative (Stahel *et al.*, 1985; Berendsen *et al.*, 1988; Canon *et al.*, 1988; Trillet *et al.*, 1989; Beiske *et al.*, 1992). In order to prevent giving tumour cells back to the patient, it is logical to purge the bone marrow for tumour cells before reinfusion. The usefulness of immunological, physical and chemical procedures have been examined for this purpose (Gordon *et al.*, 1984; Mabry *et al.*, 1985; Okabe *et al.*, 1985; Benard *et al.*, 1988; Combaret *et al.*, 1988; Vredenburgh & Ball, 1990; Humblet *et al.*, 1989; Meagher *et al.*, 1989; Elias *et al.*, 1990).

In this study the purging efficacy of immunomagnetic beads used in combination with four different monoclonal antibodies (MAbs), previously selected from a panel of 17 MAbs (Myklebust *et al.*, 1991), was tested in preclinical model experiments. The optimal procedure involved the use of a mixture of all four MAbs and two purging cycles, resulting in as high as 6 log of tumour cell depletion with an acceptable recovery of normal bone marrow progenitor cells.

Correspondence: Ø. Fodstad, Department of Tumor Biology, The Norwegian Radium Hospital, Montebello 0310 Oslo 3, Norway.
Received 16 November 1992; and in revised form 4 January 1993.

### Materials and methods

### *Bone marrow*

Human bone marrow aspirates were obtained from healthy volunteers or from patients at the Norwegian Radium Hospital with non-SCLC malignancies that were free of tumour cells in their marrow. All samples were obtained with informed consent from the donors. Ten ml of bone marrow were layered upon Lymphoprep (Nycomed, Oslo, Norway) and the mononuclear cell fraction was obtained by centrifugation at 1200 r.p.m. for 30 min. The mononuclear cells were washed once in phosphate buffered saline (PBS) before being used in the experiments.

### *Cell line*

The H-146 human SCLC cell line (Carney *et al.*, 1985), kindly provided by Dr Adi F. Gazdar, National Cancer Institute, Bethesda, MD, USA, was used in the experiments. The cells were grown as suspension cultures in RPMI 1640 medium (Gibco, Paisley, UK) supplemented with 10% foetal calf serum (FCS).

### *Monoclonal antibodies*

MOC-1 (Leij *et al.*, 1985) and MOC-31 (Leij *et al.*, 1986) antibodies were gifts from Dr L. de Leij, Groningen, The Netherlands. MOC-1 (IgG1) binds to the neural cell adhesion molecule (CD56/NCAM) (Moolenaar *et al.*, 1990), and MOC-31 (IgG2a) to an epithelial antigen (cluster 2). NrLu10 (also denoted TFS-2), an IgG2b antibody recognising a 39 kDa antigen (Okabe *et al.*, 1984), was obtained from NeoRx Corporation, Seattle, WA, USA. MLuC1 (earlier denoted MOv15), kindly provided by Dr Sylvie Menard, Milan, Italy, is an IgG1 antibody that binds to a saccharide epitope carried by neutral glycolipids, glycoproteins and mucins (Ripamonti *et al.*, 1987).

### *Immunomagnetic beads*

The immunomagnetic beads used, Dynabeads$^R$ M-450 SAM IgG ST (Dynal, Oslo, Norway), are uniform, magnetic, polystyrene beads with sheep anti mouse IgG (SAM) antibodies covalently bound to their surface. SAM bind all mouse IgG subclasses.

*Immunomagnetic separation procedure*

Mechanically dispersed tumour cells ($1 \times 10^7$), mononuclear bone marrow cells ($2 \times 10^6$), or mixes of both (total $5 \times 10^7$) were incubated in plastic tubes for 30 min at 4°C with one or more MAbs (10 μg of each) in 1 ml of RPMI 1640, supplemented with 10% FCS, 100 units of penicillin and 100 μl of streptomycin (Gibco) per ml medium. The cells were washed twice in PBS with 1% FCS before adding the immunomagnetic beads suspended in 0.5 ml of medium and then incubated at 4°C for 30 min. In the experiments with the cell line the ratio of beads to tumour cells was 50:1, and in the control experiments to test the effect on bone marrow progenitor cells the ratio of beads to nucleated bone marrow cells was also 50:1. To ensure proper contact between cells and MAbs/beads, during the incubations, the tubes were rolled on a mixer (Coulter Electronics Ltd., UK). Tumour cells were removed from the suspension by placing a flat cobalt samarium magnet to the wall of the tubes. After 1 min the suspension with remaining cells was aspirated, centrifugated at 1000 r.p.m. for 5 min, and the cells resuspended in 1 ml of medium. Remaining clonogenic tumour cells and bone marrow cells in the suspension were measured by colony-forming assays as described below. In some experiments, a repeat cycle of immunomagnetic separation was performed.

*Colony-forming assays for tumour and bone marrow progenitor cells*

The number of colony forming tumour cells was assessed in a soft agar assay (Courtenay & Mills, 1978), as previously described for malignant B cells (Kvalheim *et al.*, 1987). Briefly, soft agar cultures were set up in triplicates in 10 ml tubes by adding 0.2 ml August rat blood diluted 1 to 8, 0.2 ml of appropriately diluted bone marrow/tumour cell suspensions, and 0.6 ml of 0.5% agar (Difco Laboratories, Detroit, Michigan, USA). The tubes were incubated at 37°C in 5% $O_2$, 5% $CO_2$, and 90% $N_2$, and after 21 days of incubation colonies of more than 50 cells were counted using a Zeiss stereo microscope.

The clonogenic progenitor cells remaining in the bone marrow after immunobead separation were assessed in a modified version (Kvalheim *et al.*, 1987) of the method described by Burgess *et al.* (1977). Mononuclear BM cells were suspended at a concentration of $2 \times 10^5$ cells $ml^{-1}$ in MCCOY'S 5A medium (Gibco) containing 0.3% agar, 15% FCS, 20 ng of GM-CSF (kindly provided by Schering Plough, NJ, USA) and antibiotics. Triplicate 1 ml aliquots were cultured in 35 mm plastic dishes at 37°C in 5% $O_2$, 5% $CO_2$, and 90% $N_2$. After 14 days of incubation colonies of more than 40 cells were counted.

In the GEMM assay (Messner *et al.*, 1982), $2 \times 10^5$ mononuclear cells were suspended in 1 ml Iscove's modified Dulbecco's medium (Gibco) containing 0.8% methylcellulose, 10% FCS, 10% leucocyte conditioned medium, 30% human plasma, $5 \times 10^{-5}$ M 2-mercaptoethanol and 1 unit of erythropoietin (Cilag Ltd., Schaffhausen, Switzerland). Triplicate 1 ml aliquots were cultured and colonies counted as for the GM assay.

### Results

*Cloning efficiency of SCLC cells*

When different numbers of H-146 cells were seeded out in soft agar the number of colonies formed was proportional to the number of cells plated (Figure 1). This relationship was close to linear down to ten cells plated per tube, and the plating efficiency calculated from the slope of the curve was approximately 40% in three independent experiments. Similar curves were established in each model experiment, and the curves were used to calculate the tumour cell depletion obtained by the treatment, taking the plating efficiency in each experiment into account. Efficacy of the treatment is given as the logarithm of the number of tumour cells removed.

*Tumour cell removal*

*Efficacy of immunobeads used with single and combinations of two MAbs* Of the individual MAbs, MOC-1 and NrLu10 were the most effective in the immunomagnetic procedure, resulting in 3.7 log and 3.6 log tumour cell removal, respectively (Table I). The corresponding value for MLuC1 was 2.6 log, compared to 2.1 log for MOC-31. When the procedure was performed twice, a significant increase in efficacy was obtained for MOC-1 (4.4 log depletion) and for MLuC1 (4.2 log depletion), whereas the values with the other two MAbs increased only slightly (Table I).

Since it is known that tumour-associated antigens commonly are heterogeneously expressed on tumour cells, we wanted to test different combinations of two MAbs. As can

**Figure 1** Sensitivity and reproducibility of the soft-agar assay of clonogenic tumour cells. Different cell numbers of the human small cell lung cancer line H-146 were seeded out in the assay as described in Materials and methods. Each point represents the mean of results obtained in three independent experiments, each in triplicate. Bars indicate s.d.

**Table I** Efficacy of sheep anti-mouse (SAM)-coated paramagnetic particles (immunobeads) in removing H-146 small cell lung cancer cells incubated with different primary antibodies, individually and in combinations of two

| | Log tumour cell depletion[a] (mean)[b] | | | | | | | | | |
|---|---|---|---|---|---|---|---|---|---|---|
| | Individual MAbs | | Combination of two MAbs | | | | | | | |
| | | | One cycle of elimination | | | | Two cycles of elimination | | | |
| *Mab* | *One cycle of elimination* | *Two cycles of elimination* | *MOC-1* | *MOC-31* | *NrLu10* | *MLuC1* | *MOC-1* | *MOC-31* | *NrLu10* | *MLuC1* |
| MOC-1 | 3.7 | 4.4 | – | 3.0 | 3.9 | 3.6 | – | 4.1 | 4.1 | 4.2 |
| MOC-31 | 2.1 | 2.5 | 3.0 | – | 3.7 | 3.8 | 4.1 | – | 4.6 | 4.7 |
| NrLu10 | 3.6 | 3.9 | 3.9 | 3.7 | – | 4.0 | 4.1 | 4.6 | – | 4.2 |
| MLuC1 | 2.6 | 4.2 | 3.6 | 3.8 | 4.0 | – | 4.2 | 4.7 | 4.2 | – |

H-146 cells were incubated for 30 min at 4°C with MAbs (10 μg $ml^{-1}$ of each) and then for 30 min with SAM-M-450 Dynabeads at a ratio beads to tumour cells of 50:1. [a]Calculated from the number of colonies counted, taking the plating efficiency into account, and determined as the logarithm of the number of cells depleted by the treatment. [b]Mean of the results obtained in 2–3 independent soft agar experiments, each performed in triplicate.

be seen from Table I, the different combinations gave log tumour cell depletions in the range of 3.0–4.7. In most cases the combinations were not more effective than the most effective single antibody, except in some experiments involving MOC-31 and MLuC1. The results were clearly improved by performing the elimination procedure twice. The overall best results were obtained with combinations of either MOC-31 and MLuC1 or NrLu10 and MOC-31, resulting in 4.6–4.7 log tumour cell depletion.

*Combinations of three and four MAbs* In the next set of experiments, combinations of three (MOC-1 + MOC-31 + NrLu10) and four (MOC-1 + MOC-31 + NrLu10 + MLuC1) antibodies were tested. As can be seen from the results presented in Table II, neither of the combinations was more effective than the most active single antibodies (Table I) when only one cycle of immunomagnetic removal was used. With two cycles, however, the tumour cell depletion with a combination of three MAbs increased to 4.7 log, a comparable efficacy to that of the most effective 2-MAb combination. This value was further increased to 5.1 log when an additional incubation with the antibodies was introduced before the second cycle of immunomagnetic separation (Table II). The most effective treatment was obtained with two elimination cycles involving the combination of four antibodies. Thus, the mean log tumour cell depletion obtained in three different experiments was 5.6 log, ranging from 5.3 to at least 6 logs. In this case, the additional incubation with the MAbs did not improve the results.

With the encouraging results obtained, the combinations of three and four MAbs were also tested in the more clinically relevant situation with H-146 tumour cells added to fresh human bone marrow at a ratio of 1:10. In repeat experiments, a mean log tumour cell depletion of 4.8 was obtained with a mixture of three MAbs (Table III). In one of the experiments, only 10 out of $1 \times 10^6$ tumour cells plated remained in the bone marrow after purging, as calculated from the number of colonies formed in soft agar. Even more encouraging, with the combination of four MAbs no colony formation in soft agar was observed with a tumour cell/bone marrow mixture that contained $1 \times 10^6$ SCLC cells before purging. Since the same result was obtained in three independent experiments, it can be concluded that with the 4 MAb combination at least 6 log tumour cell depletion from the bone marrow can be achieved, a result that is even better than when the procedure was performed without the presence of bone marrow cells.

*Survival of progenitor bone marrow cells*

The effect of the immunomagnetic procedure on the survival of normal bone marrow progenitor cells was assessed (Table IV). With single antibodies, the fraction of surviving CFU-GM differed with the antibodies from 80 to 49%, the lowest value was seen in experiments with MLuC1. With three and four MAb combinations, the fraction of surviving CFU-GM was 68% and 52%, respectively. The combinations were also tested in the CFU-GEMM assay, where it was found that 70–80% of the progenitor cells had survived the immunomagnetic treatment. The results indicate that the immunomagnetic purging procedure involving combinations of MAbs can be used safely for purging purposes in patients.

### Discussion

A magnetic procedure was developed for removing SCLC cells from human bone marrow. We have demonstrated that by using a combination of four antibodies and immunomagnetic sheep anti-mouse IgG beads, a 6-log tumour cell separation could be achieved, as determined by a reproducible clonogenic soft agar assay. The procedure reduced the survival of normal bone marrow progenitor cells to only a limited extent, showing that the procedure can be safely used in a clinical setting.

Heterogeneity of tumour cell surface antigen expression suggests that a cocktail of MAbs may be necessary to achieve effective removal of tumour cells. This was confirmed in the present work, as the best results were obtained with a combination of all four MAbs. The MAbs were selected because of their ability to bind to small cell lung cancer cells, and not to normal cells in bone marrow and peripheral blood (Myklebust *et al.*, 1991). Also, the usefulness of the same antibodies for detecting bone marrow metastases in small cell lung cancer patients has recently been demonstrated (Beiske *et al.*, 1992). We have found that the NrLu10 and MOC-31 antibodies bind to the same antigen, but to different epitopes (unpublished data). However, they differ in their ability to

**Table II** Efficacy of immunobeads in eliminating H-146 cells incubated with combinations of three or four anti-small cell lung cancer antibodies. Effect of repeat immunomagnetic removal with and without additional incubation with primary antibodies

| *MAbs* | *Number of experiments* | *Cycle of Elimination* | *Cycle of MAbs* | *Log tumour cell depletion (mean)* | *Log tumour cell depletion (range)* |
|---|---|---|---|---|---|
| MOC-1 + MOC-31 + NrLu10 | 3 | 1 | 1 | 3.8 | 3.4– 4.1 |
| | 3 | 2 | 1 | 4.7 | 4.4– 5.1 |
| | 2 | 2 | 2 | 5.1 | 5.0– 5.2 |
| MOC-1 + MOC-31 + NrLu10 + MLuC1 | 2 | 1 | 1 | 3.3 | 2.7– 3.8 |
| | 3 | 2 | 1 | 5.6 | 5.3–>6.0 |
| | 1 | 2 | 2 | 5.4 | – |

Treatment and calculations as in Table I.

**Table III** Removal of H-146 cells from fresh human bone marrow using immunobeads and a mixture of three or four monoclonal antibodies

| *MAbs* | *Number of experiments* | *Tumour cells plated* | *Tumour cells remaining (mean)* | *Log tumour cell depletion (mean)* | *Log tumour cell depletion (range)* |
|---|---|---|---|---|---|
| MOC-1 + MOC-31 + NrLu10 | 2 | $10^5$ | 3 | 4.8 | 4.5–5.0 |
| | | $10^6$ | 10 | | |
| MOC-1 + MOC-31 + NrLu10 + MLuC1 | 3 | $10^6$ | 0 | >6.0 | >6.0 |

H-146 cells mixed with bone marrow cells (ratio 1:10) were incubated with the MAbs (10 $\mu g\ ml^{-1}$ of each) and then with SAM-M-450 Dynabeads at a ratio tumour cells to beads of 1:50. Two purging cycles were used. Calculations as in Table I.

**Table IV** Survival of normal bone marrow progenitor cells after treatment[a] with monoclonal antibodies and immunobeads

| | *CFU-GM* | | | *CFU-GEMM* | | |
|---|---|---|---|---|---|---|
| *MAbs* | *No. of colonies (mean)*[b] | *Fraction of control*[c] *(%)* | *(range)* | *No. of colonies (mean)*[b] | *Fraction of control*[c] *(%)* | *(range)* |
| None | 111 | 80 | 69–100 | nt | – | |
| MOC-1 | 106 | 67 | 53– 75 | nt | – | |
| MOC-31 | 97 | 86 | 70– 99 | nt | – | |
| NrLu10 | 90 | 67 | 59– 75 | nt | – | |
| MLuC1 | 69 | 49 | 32– 69 | nt | – | |
| MOC-1 + MOC-31 + NrLu10 | 96 | 68 | 61– 83 | 137 | 70 | 67–74 |
| MOC-1 + MOC-31 + NrLu10 + MLuC1 | 70 | 49 | 45– 56 | 135 | 80 | 64–94 |

[a]Two × $10^5$ nucleated bone marrow cells were incubated with the MAbs (10 μg $ml^{-1}$ of each), and then with immunobeads at a ratio beads to mononuclear cells of 50:1, in both cases for 30 min at 4°C. [b]Mean of the results in three independent experiments, each in triplicate. [c]Fraction of remaining colonies calculated relative to the number of colonies in untreated control cultures.

detect contaminating SCLC cells in fresh bone marrow aspirates (Beiske *et al.*, 1992), and as suggested by the present results, both antibodies should be included in a cocktail recommended for clinical use, together with MOC-1 and MLuC1 that recognise distinct antigens (Moolenaar *et al.*, 1990; Ripamonti *et al.*, 1987).

To limit the need for fresh human bone marrow, the removal experiments with individual and combinations of two MAbs were performed on a suspension of H-146 cells only, as previous experience has shown that such experiments closely predict results obtained when tumour cells are mixed with normal bone marrow cells. In accordance with this, the present procedure proved to be equally effective when H-146 tumour cells were mixed with bone marrow cells at a ratio of 1:10. Thus, the optimal combinations of four MAbs resulted in a mean log tumour cell removal of 5.6 in the absence, and >6.0 in the presence of bone marrow cells. In experiments with MOC-31 antibody no difference in log tumour cell removal was found between 1:10 and 1:100 mixes of SCLC and marrow cells (not shown). However, limited access to human bone marrow and the high efficacy of the purging method prevented further testing at a low tumour to bone marrow cell ratio. The validity of calculating log tumour cell removal is dependent on a close to linear relationship between the number of tumour cells plated and the number of soft agar colonies formed. This requirement was met with the H-146 cell line, which showed a high and reproducible plating efficiency in the clonogenic assay.

Several reports have described methods to eliminate SCLC cells from bone marrow (Gordon *et al.*, 1984; Mabry *et al.*, 1985; Okabe *et al.*, 1985; Benard *et al.*, 1988; Vredenburgh & Ball, 1990; Humblet *et al.*, 1989; Meagher *et al.*, 1989; Elias *et al.*, 1990). Vredenburgh & Ball (1990) obtained 4–5 log separation using immunomagnetic beads and three different antibodies, administered at a relatively high concentration, whereas the results of Elias *et al.* (1990) were less satisfactory (2.4–2.6 log). In both cases, two types of immunobeads had to be used because their tumour-associated primary antibodies were of both IgG and IgM isotypes. The results reported for other depletion procedures show varying degrees of efficacy and toxicity to normal bone marrow cells (Gordon *et al.*, 1984; Mabry *et al.*, 1985; Okabe *et al.*, 1985; Benard *et al.*, 1988; Meagher *et al.*, 1989), the best results (4–5 log depletion) obtained by Humblet *et al.* (1989). The latter investigators combined immunological and pharmacological methods, and their regime was relatively toxic to bone marrow progenitor cells.

Altogether, the results in the present work were better than any of those previously reported, probably reflecting both the efficacy of immunomagnetic procedures (Kiesel *et al.*, 1987; Kvalheim *et al.*, 1988) and the properties of the antibodies used. In agreement with the experience of Vredenburgh & Ball (1990), the best results were obtained with two purging cycles at a 50:1 ratio of beads to tumour cells. The procedure had only minor effects on cell survival in the bone marrow progenitor cell assays. Some bone marrow specimens used for these studies were obtained from patients with other types of malignancies but without bone marrow involvement. In a few of these cases, the proliferative capacity of the progenitor cells was relatively low. This did not influence the results, except that with MLuC1 used alone an unpredictable variation in the number of surviving CFU-GMs was observed in some experiments.

High dose chemotherapy followed by ABMT in SCLC patients has been used by several investigators (Humblet *et al.*, 1987; Symann *et al.*, 1989; Souhami *et al.*, 1989; Williams *et al.*, 1989; Marangolo *et al.*, 1989; Nomura *et al.*, 1990; Lazarus *et al.*, 1990; Gomm *et al.*, 1991), but no improvement in survival, compared to patients treated with standard chemotherapy, has been reported. Several factors may have contributed to the reported limited benefit of ABMT, including a less than optimal patient selection with regards to stage of the disease, age, response to induction therapy, and timing and intensity of the ablative treatment. Moreover, with the development of sensitive diagnostic techniques it has become increasingly clear that a high number of SCLC patients have contaminating tumour cells in their bone marrow (Stahel *et al.*, 1985; Berendsen *et al.*, 1988; Canon *et al.*, 1988; Trillet *et al.*, 1989; Beiske *et al.*, 1992). It should also be noted that the detection of tumour cells in bone marrow is based on studies of small volume aspirates, whereas about 1 l of bone marrow is harvested for ABMT. Thus, the risk of tumour cell contamination of the transfused marrow may be higher than indicated by studying bone marrow aspirates.

One argument against ABMT in SCLC has been that several patients have relapsed locally in the lungs. It cannot be excluded, however, that these relapses are associated with i.v. transfusion of contaminated bone marrow cells. The reinfused cells will meet the first capillary bed in the lungs, and it is well known that interaction between tumour cells and host tissues favours tumour growth in orthotopic sites, in this case represented by the lungs. Furthermore, the effect of tumour cell trapping in the lungs is also exemplified by the finding of Glorieux *et al.* (1986) that a patient with neuroblastoma and another with Burkitts lymphoma developed diffuse carcinomatosis in the lungs after ABMT.

In cancer types where patients undergoing ABMT are at risk for having contaminating tumour cells in the bone marrow, it seems logical that the marrow should be purged before transfusion. Although the value of tumour cell purging has not been definitely proven, recent evidence indicates that effective removal of lymphoma cells from the marrow has a significant effect on the clinical outcome (Gribben *et al.*, 1991). We have previously developed highly effective immunomagnetic methods which are currently in routine clinical use in patients with B and T-cell malignancies (Kvalheim *et al.*, 1988; Wang *et al.*, 1992). The similar method described here for purging SCLC has been shown to be equally efficacious in tumour cell depletion from bone marrow, and the method is recommended for use in conjunction with ABMT in selected groups of SCLC patients.

Arne T. Myklebust is a Fellow of the Norwegian Cancer Society. We thank Aslak Godal for help in purification of the MAbs, Gunnar Kvalheim for valuable advice, and Frances Jaques for excellent secretarial assistance. We appreciate the help of Erling Jakobsen in obtaining bone marrow aspirates.

This work was supported by The Norwegian Cancer Society and the Blix legacy.

### References

ALBAIN, K.S., CROWLEY, J.J. & LIVINGSTON, R.B. (1991). Long-term survival and toxicity in small cell lung cancer. *Chest*, **99**, 1425–1432.

BEISKE, K., MYKLEBUST, A.T., AAMDAL, S., LANGHOLM, R., JAKOBSEN, E. & FODSTAD, Ø. (1992). Detection of bone marrow metastases in small cell lung cancer patients. Comparison of immunologic and morphologic methods. *Am. J. Pathol.*, **141**, 531–538.

BENARD, J., BETTAN-RENAUD, L., GAVOILLE, A., PICO, J.-L., BEAUJEAN, F., LOPEZ, M. & RIOU, G. (1988). In vitro chemical eradication of small cell lung cancer: application in autologous bone marrow transplantation. *Eur. J. Cancer Clin. Oncol.*, **24**, 1561–1566.

BERENDSEN, H.H., DE LEIJ, L., POSTMUS, P.E., TER HAAR, J.G., POPPEMA, S. & THE, T.H. (1988). Detection of small cell lung cancer metastases in bone marrow aspirates using monoclonal antibody directed against neuroendocrine differentiation antigen. *J. Clin. Pathol.*, **41**, 273–276.

BURGESS, A.W., WILSON, E.M.A. & METCALF, D. (1977). Stimulation by human placental conditioned medium of hemopoietic colony formation by human marrow cells. *Blood*, **49**, 573–583.

CANON, J.L., HUMBLET, Y., LEBACQ-VERHEYDEN, A.M., MANOUVRIEZ, P., BAZIN, H., RODHAIN, J., PRIGNOT, J. & SYMANN, M. (1988). Immunodetection of small cell lung cancer metastases in bone marrow using three monoclonal antibodies. *Eur. J. Cancer Clin. Oncol.*, **24**, 147–150.

CARNEY, D.N., GAZDAR, A.F., BEPLER, G., GUCCION, J.G., MARANGOS, P.J., MOODY, T.W., ZWEIG, M.H. & MINNA, J.D. (1985). Establishment and identification of small cell lung cancer cell lines having classic and variant features. *Cancer Res.*, **45**, 2913–2923.

COURTENAY, V.D. & MILLS, J. (1978). An *in vitro* colony assay for human tumours grown in immune-suppressed mice and treated *in vivo* with cytotoxic agents. *Br. J. Cancer*, **37**, 261–268.

ELIAS, A.D., PAP, S.A. & BERNAL, S.D. (1990). Purging of small cell lung cancer-contaminated bone marrow by monoclonal antibodies and magnetic beads. *Prog. Clin. Biol. Res.*, **333**, 263–275.

GLORIEUX, P., BOUFFET, E., PHILIP, I., BIRON, P., HOLZAPFEL, L., FLORET, D., BOUVIER, R., VITREY, D., PINKERTON, R., BRUNAT-MENTIGNY, M. & PHILIP, T. (1986). Metastatic interstitial pneumonitis after autologous bone marrow transplantation. A consequence of reinjection of malignant cells? *Cancer*, **58**, 2136–2139.

GOMM, S.A., THATCHER, N., CUTHBERT, A., CHANG, J., BURMESTER, H., HALL, P. & CARROLL, K.B. (1991). High dose combination chemotherapy with ifosfamide, cyclophosphamide or cisplatin, mitomycin C and mustine with autologous bone marrow suppoort in advanced non-small cell lung cancer. A phase I/II study. *Br. J. Cancer*, **63**, 293–297.

GORDON, L.I., ROSEN, S.T., VRIESENDORP, H.M., KIES, M.S. & KUCUK, O. (1984). Separation of clonogenic tumor cells from small cell lung cancer bone marrow and small cell lung cancer cell lines. *Cancer Res.*, **44**, 5404–5408.

GRIBBEN, J.G., FREEDMAN, A.S., NEUBERG, D., ROY, D.C., BLAKE, K.W., WOO, S.D., GROSSBARD, M.L., RABINOWE, S.N., CORAL, F., FREEMAN, G.J., RITZ, J. & NADLER, L.M. (1991). Immunologic purging of marrow assessed by PCR before autologous bone marrow transplantation for B-cell lymphoma. *N. Engl. J. Med.*, **325**, 1525–1533.

HUMBLET, Y., FEYENS, A.M., SEKHAVAT, M., AGALIOTIS, D., CANON, J.L. & SYMANN, M.L. (1989). Immunological and pharmacological removal of small cell lung cancer cells from bone marrow autografts. *Cancer Res.*, **49**, 5058–5061.

HUMBLET, Y., SYMANN, M., BOSLY, A., DELAUNOIS, L., FRANCIS, C., MACHIELS, J., BEAUDUIN, M., DOYEN, C., WEYNANTS, P., LONGUEVILLE, J. & PRIGNOT, J. (1987). Late intensification chemotherapy with autologous bone marrow transplantation in selected small-cell carcinoma of the lung: a randomized study. *J. Clin. Oncol.*, **5**, 1864–1873.

KIESEL, S., HAAS, R., MOLDENHAUER, G., KVALHEIM, G., PEZZUTTO, A. & DÖRKEN, B. (1987). Removal of cells from malignant B-cell line from bone marrow with immunomagnetic beads and with complement and immunoglobulin switch variant mediated cytolysis. *Leuk. Res.*, **11**, 1119–1125.

KVALHEIM, G., FODSTAD, Ø., PIHL, A., NUSTAD, K., PHARO, A., UGELSTAD, J. & FUNDERUD, S. (1987). Elimination of B-lymphoma cells from human bone marrow: model experiments using monodisperse magnetic particles coated with primary monoclonal antibodies. *Cancer Res.*, **47**, 846–851.

KVALHEIM, G., SØRENSEN, O., FODSTAD, Ø., FUNDERUD, S., KIESELS, S., DÖRKEN, B., NUSTAD, K., JAKOBSEN, E., UGELSTAD, J. & PIHL, A. (1988). Immunomagnetic removal of B-lymphoma cells from human bone marrow: a procedure for clinical use. *Bone Marrow Transplant.*, **3**, 31–41.

LAZARUS, H.M., SPITZER, T.R. & CREGER, R.J. (1990). Phase i trial of high-dose etoposide, high-dose cisplatin, and reinfusion of autologous bone marrow for lung cancer. *Am. J. Clin. Oncol.*, **13**, 107–112.

LEIJ, L.D., POPPEMA, S., NULEND, J.K., HAAR, A.T., SCHWANDER, E., EBBENS, F., POSTMUS, P.E. & THE, T.H. (1985). Neuroendocrine differentiation antigen on human lung carcinoma and Kulchitski cells. *Cancer Res.*, **45**, 2192–2200.

LEIJ, L.D., POSTMUS, P.E., POPPEMA, S., ELEMA, J.D. & THE, T.H. (1986). The use of monoclonal antibodies for the pathological diagnosis of lung cancer. In *Lung Cancer: Basic and Clinical Aspects*, Hansen, H.H. (ed.), p. 31. Martinus Nijhoff Publishers: Boston.

MABRY, M., SPEAK, A., GRIFFIN, J.D., STAHEL, R.A. & BERNAL, S.D. (1985). Use of SM-1 monoclonal antibody and human complement in selective killing of small cell carcinoma of the lung. *J. Clin. Invest.*, **75**, 1690–1695.

MARANGOLO, M., ROSTI, G., AMADORI, D., LEONI, M., ARDIZZONE, A., FIORENTINI, G., CRUCIANI, G., TIENGHI, A., RAVAIOLI, A., SEBASTIANI, L., TURCI, D., COTIGNOLI, T., ARGNANI, M., FLAMINI, E. & ROSSO, R. (1989). High-dose etopside and autologous bone marrow transplantation as intensification treatment in small cell lung cancer: a pilot study. *Bone Marrow Transplant.*, **4**, 405–408.

MEAGHER, R.C., ROTHMAN, S.A., PAUL, P., KOBERNA, P., WILLMER, C. & BAUCCO, P.A. (1989). Purging of small cell lung cancer cells from human bone marrow using ethiofos (WR-2721) and light-activated merocyanine 540 phototreatment. *Cancer Res.*, **49**, 3637–3641.

MESSNER, H.A., JAMAL, N. & IZAGUIRRE, E. (1982). The growth of large megakaryocyte colonies from human bone marrow. *J. Cell. Physiol. (Suppl.)*, **1**, 45–51.

MOOLENAAR, C.E.C.K., MULLER, E.J., SCHOL, D.J., FIGDOR, C.G., BOCK, E., BITTER-SAUERMANN, D. & MICHALIDES, R.J.A.M. (1990). Expression of neural cell adhesion molecule-related sialoglycoprotein in small cell lung cancer and neuroblastoma cell lines H69 and CHP-212. *Cancer Res.*, **50**, 1102–1106.

MYKLEBUST, A.T., BEISKE, K., PHARO, A., DAVIES, C.DE.L., AAMDAL, S. & FODSTAD, Ø. (1991). Selection of anti-SCLC antibodies for diagnosis of bone marrow metastasis. *Br. J. Cancer (Suppl. XII)*, **63**, 49–53.

NOMURA, F., SHIMOKATA, K., SAITO, H., WATANABE, A., SAKA, H., SAKAI, S., KODERA, Y. & SAITO, H. (1990). High dose chemotherapy with autologous bone marrow transplantation for limitid small cell lung cancer. *Jpn. J. Clin. Oncol.*, **20**, 94–98.

OKABE, T., KAIZU, T., FUJISAWA, M., WATANABE, J., KOJIMA, K., YAMASHITA, T. & TAKAKU, F. (1984). Monoclonal antibodies to surface antigens of small cell carcinoma of the lung. *Cancer Res.*, **44**, 5273–5278.

OKABE, T., KAIZU, T., OZAWA, K., URABE, A. & TAKAKU, F. (1985). Elimination of small cell lung cancer cells *in vitro* from human bone marrow by a monoclonal antibody. *Cancer Res.*, **45**, 1930–1933.

RIPAMONTI, M., CANEVARI, S., MENARD, S., MEZZANZANICA, D., MIOTTI, S., ORLANDI, R., RILKE, F., TAGLIABUE, E. & COLNAGHI, M.I. (1987). Human carcinoma cell lines xenografted in athymic mice: biological and antigenic characteristics of an intra-abdominal model. *Cancer Immunol. Immunother.*, **24**, 13–18.

SOUHAMI, R.L., HAJICHRISTOU, H.T., MILES, D.W., EARL, H.M., HARPER, P.G., ASH, C.M., GOLDSTONE, A.H., SPIRO, S.G., GEDDES, D.M. & TOBIAS, J.S. (1989). Intensive chemotherapy with autologous bone marrow transplantation for small-cell lung cancer. *Cancer Chemother. Pharmacol.*, **24**, 321–325.

STAHEL, R.A., MABRY, M., SKARIN, A.T., SPEAK, J. & BERNAL, S.D. (1985). Detection of bone marrow metastasis in small cell lung cancer by monoclonal antibody. *J. Clin. Oncol.*, **3**, 455-455.

SYMANN, M., HUMBLET, Y. & BOSLY, A. (1989). Clinical and biological aspects of intensive chemotherapy with autologous bone marrow grafts in small-cell bronchial cancer. *Bull. Mem. Acad. R. Med. Belg.*, **144**, 213-221.

TRILLET, V., REVEL, D., COMBARET, V., FAVROT, M., LOIRE, R., TABIB, A., PAGES, J., JACQUEMET, P., BONMARTIN, A., MORNEX, J.F., CORDIER, J.F., PINET, F., AMIEL, M. & BRUNE, J. (1989). Bone marrow metastases in small cell lung cancer: detection with magnetic resonance imaging and monoclonal antibodies. *Br. J. Cancer*, **60**, 83-88.

VREDENBURGH, J.J. & BALL, E.D. (1990). Elimination of small cell carcinoma of the lung from human bone marrow by monoclonal antibodies and immunomagnetic beads. *Cancer Res.*, **50**, 7216-7220.

WANG, M.Y., KVALHEIM, G., KVALØY, S., BEISKE, K., JAKOBSEN, E., WIJDENES, J., PIHL, A. & FODSTAD, Ø. (1992). An effective immunomagnetic method for bone marrow purging in T cell malignancies. *Bone Marrow Transplant.*, **9**, 319-323.

WILLIAMS, S.F., BITRAN, J.D., HOFFMAN, P.C., ROBIN, E., FULLEM, L., BESCHORNER, J., GOLICK, J. & GOLOMB, H.M. (1989). High-dose, multiple-alkylator chemotherapy with autologous bone marrow reinfusion in patients with advanced non-small cell lung cancer. *Cancer*, **63**, 238-242.